# Machine Learning for Communications

**DOI:** 10.3390/e23070831

**Published:** 2021-06-29

**Authors:** Vaneet Aggarwal

**Affiliations:** School of Industrial Engineering and School of Electrical and Computer Engineering, Purdue University, West Lafayette, IN 47907, USA; vaneet@purdue.edu

Due to the proliferation of applications and services that run over communication networks, ranging from video streaming and data analytics to robotics and augmented reality, tomorrow’s networks will be faced with increasing challenges resulting from the explosive growth of data traffic demand with significantly varying performance requirements. This calls for more powerful, intelligent methods to enable novel network design, deployment, and management. To realize this vision, there is an increasing need to leverage recent developments in machine learning (ML), as well as other artificial intelligence (AI) techniques, and fully integrate them into the design and optimization of communication networks.

In this editorial, we will first summarize the key problem structures in communication systems where machine learning solutions have been used. Then, we will describe the areas where there are gaps in learning algorithms for their optimal applications to communication systems.

In the following, we will describe the different problem structures in communication systems, which can be solved by ML approaches. 

**Parametric Optimization with Deep Neural Networks:** The formulation of parametric optimization is given as follows.

(1) $$\begin{array}{ccc} x^{*}\left(\theta \right) & = & \arg\min_{x}f\left(x,\theta \right) \end{array}$$

In this problem, the aim is to represent the solution to an entire family of problems (for all θ). One approach for solving such problems is to use a Deep Neural Network with θ as an input and $x^{*}\left(\theta \right)$ as the output. Using a certain values of θ, the optimization problem can be solved and these values make the training data for the neural network. The trained neural network is then used to obtain $x^{*}\left(\theta \right)$ for all θ. Such approaches has been used for beamforming [1] as well as power control [2]. In these problems, the channel coefficients or the signal-to-noise ratio of the links are the parameters θ based on which optimal beamforming vectors or power control solution needs to be calculated.

We note that even for a given θ, finding $x^{*}\left(\theta \right)$ maybe a hard problem which limits obtaining enough training data for the problem. Recently, the authors of [2] proposed an approach where θ is sampled, and a single step along the gradient of the objective function is taken. This allows more flexibility as the optimization problem do not need to be fully solved for the training examples. Such an approach has been validated on power control problems by the authors of [2]. While such direction has great empirical evidence, convergence rates to global optimal $x^{*}\left(\theta \right)$ with samples is an open problem, to the best of our knowledge. 

**Reinforcement Learning for Combinatorial Optimization:** Many problems in communication systems require combinatorial optimization, e.g., routing optimization, scheduling, and resource allocation [3]. Many combinatorial problems are NP-hard, and thus key approaches for such problems have been approximation algorithms, hand-crafted heuristics, or meta-heuristics [4]. The combinatorial optimization problem can be formulated as follows: Let V be a set of elements and f:V→R be a cost function. Combinatorial optimization problem aims to find an optimal value of the function *f* and any corresponding optimal element that achieves that optimal value on the domain. One of the emerging recent trends is to solve combinatorial optimization problems by using a reinforcement learning approach. In this approach, the combinatorial optimization problem is formulated as a Markov Decision Process (MDP). The state encodes the current solution, and the action describes the modification of the current solution. The reward is given by the change in objective with the modification. The exact state and action encoding depends on the problem and the approach used. A recent survey of the different approaches based on reinforcement learning to combinatorial optimization are presented in [5]. We note that combinatorial optimization approaches using reinforcement learning have been used in communications to find efficient encoding designs [6,7,8].

**Reinforcement Learning for Dynamic Resource Management:** In the presence of dynamic job arrivals, online resource management of computing and communication resources become important. Consider an example of a single queue which is serving different types of customers. The overall objective is to minimize weighted latency of the different types of customers, where the queue needs to decide which of the customer request to be processed next. This can be modeled as a Markov decision process with the state as the vector composed of the queue length of each type of customer, action is to choose which of the customer request to be processed next, and the cost (negative of reward) is the weighted latency of the served customer. The current action impacts the next state and leads to a dynamic system. In networking problems, such scheduling problems occur at all layers, which make the use of reinforcement learning important in networking problems. In particular, modern networks such as Internet of Things (IoT), Heterogeneous Networks (HetNets), and Unmanned Aerial Vehicle (UAV) network become more decentralized, ad-hoc, and autonomous in nature. Network entities such as IoT devices, mobile users, and UAVs need to make local and autonomous decisions, e.g., spectrum access, data rate selection, transmit power control, and base station association, to achieve the goals of different networks including, e.g., throughput maximization, and energy consumption minimization [9]. This has led to widespread use of reinforcement learning in networking applications, see [9] for a detailed survey. Some of the applications include traffic engineering [10], caching [11], queue management [12], video streaming [13], software-defined networks [14]. In addition to wireless networks, reinforcement learning for dynamic resource management has been widely used in transportation networks, e.g., vehicle routing and dispatch [15,16,17], freight scheduling [18], and traffic signal control [19].

We will now describe some of the areas where novel learning-based solutions are needed, which have applications in communication research. 

**Joint Decision of Multiple Agents:** Communication systems consist of multiple decision makers in the system, e.g., multiple base stations. With multiple decision makers, multiple challenges arise. One of them is that the joint decision requires joint state and joint action space of the users. However, this is computationally prohibitive. In order to deal with this challenge, multiple approaches have been proposed. One of the approaches is an approximation of cooperative multi-agent reinforcement leaning by a mean-field control (MFC) framework, where the approximation error is shown to be of $O(1/\sqrt{N})$ for *N* agents [20]. Another approach is the use of decentralizable algorithms, which aim to do centralized training and decentralized execution [21,22,23]. Further, there is a distributed approach which introduces communication among agents during execution [24,25]. Even though multiple approaches have been investigated, efficient complexity-performance-communication tradeoff is an important research problem. 

**Multi-objective Optimization:** Many realistic applications have multiple objectives, e.g., capacity and power usage in the communication system [26,27], latency and energy consumption [28], efficiency and safety in robotic systems [29]. Further, the overall aim is to optimize a non-linear function of the different objectives. In this setup, standard reinforcement learning approaches do not work since the non-linear objective function looses the additive structure, and thus the Bellman’s Equation does not work anymore in this setting [30]. Recently, this problem has been studied, where guarantees for model-based algorithm and model-free algorithm have been studied in [30,31], respectively. The approaches have been applied to cellular scheduling, traffic engineering, and queue scheduling problems. However, the research on this direction is still in its infancy, and scalable algorithms with better guarantees need investigation. 

**Constraints in Decision Making:** Most communication systems have constraints, e.g., power, latency, etc. Consider a wireless sensor network where the devices aim to update a server with sensor values. At time *t*, the device can choose to send a packet to obtain a reward of 1 unit or to queue the packet and obtain 0 reward. However, communicating a packet results in $p_{t}$ power consumption. At time *t*, if the wireless channel condition, $s_{t}$, is weak and the device chooses to send a packet, the resulting instantaneous power consumption, $p_{t}$, is high. The goal is to send as many packets as possible while keep the average power consumption, $\sum_{t=1}^{T}p_{t}/T$, within some limit, say *C*. This environment has state $\left(s_{t},q_{t}\right)$ as the channel condition and queue length at time *t*. To limit the power consumption, the agent may choose to send packets when the channel condition is good or when the queue length grows beyond a certain threshold. The agent aims to learn the policies in an *online manner* which requires efficiently balancing exploration of state-space and exploitation of the estimated system dynamics. Similar to the example above, many applications require to keep some costs low while simultaneously maximizing the rewards [32]. Some attempts to use constrained reinforcement learning approaches to communication problems can be seen in [12,33,34].

The problem setup, where the system dynamics are known, is extensively studied [32]. For a constrained setup, the optimal policy is possibly stochastic [32,35]. In the domain where the agent learns the system dynamics and aims to learn good policies online, there has been work where to show asymptotic convergence to optimal policies and regret guarantees for infinite horizon [36,37,38], as well as episodic MDPs [39,40]. Recently, guarantees for policy-gradient based approaches have been studied [41,42]. In addition, peak constraints have also been studied for convergence guarantees [43]. Further, algorithms with use of deep learning architectures have been studied [12,44]. Scalable algorithms with better guarantees in presence of constraints still need more investigation.

**Adaptivity to changes in the environment:** Most existing works on reinforcement learning consider a stationary environment and aim to find or be comparable to an optimal policy. In many applications, however, the environment is far from being stationary. As an example, network demands have diurnal patterns [45]. With dynamic changes in the environment, the strategies need to adapt. There has been two key approaches to measure non-stationarity of the environment. The first is where there are *L* changes in the system, and another is where the total amount of variation in the MDP is bounded by Δ. Different algorithms have been proposed to optimize the dynamic regret in this setup, with different amounts of information on *L* and Δ, for a comprehensive set of algorithms from regret perspective the reader is referred to [46]. Ideally, we require an adaptive algorithm that works without the knowledge of *L* and Δ, while achieving optimal regret bounds. Such algorithms have been shown in the episodic MDPs in tabular and linear cases [46]. There are partial results for infinite-horizon tabular case, while the proposed algorithm is not scalable. This is because the proposed algorithm opens multiple instances of base algorithms which increases the complexity of the approach. Recently, there has been an approach based on change point detection on the experience tuples to detect the change in MDPs [47], which has been applied to a sensor energy management problem and a traffic signal control problem in [47], and extended to adapt to diurnal patterns in demand of ride-sharing services in [48,49]. However, theoretical guarantees for such an approach are open.
